# Differential risk signals of cutaneous adverse drug reactions among β-lactam antibiotics: ceftriaxone shows increased signals for SJS/TEN in clinical and real-world cohorts

**DOI:** 10.3389/fmed.2026.1875934

**Published:** 2026-09-03

**Authors:** Leonard von Grundherr, Henrike Alexandra Faesser, Andreas Recke, Ralf J. Ludwig, Katharina Boch, Philip Curman

**Affiliations:** 1 Lübeck Institute of Experimental Dermatology, University of Lübeck, Lübeck, Germany; 2 Department of Dermatology, University of Lübeck, Lübeck, Germany; 3 Department of Medicine III, Pulmonology, University-Hospital Schleswig-Holstein, Lübeck, Germany; 4 Comprehensive Center for Inflammation Medicine, University-Hospital Schleswig-Holstein, Lübeck, Germany; 5 Department of Dermatology and Allergology, UniversityHospital Munich, LMU, Munich, Germany; 6 Department of Medical Epidemiology and Biostatistics, Karolinska Institutet, Stockholm, Sweden; 7 Department of Medicine (Solna), Division of Dermatology and Venereology, Karolinska Institutet, Stockholm, Sweden; 8 Dermato-Venereology Clinic, Karolinska University Hospital, Stockholm, Sweden

**Keywords:** adverse drug reactions, clinical cohort, epidermal necrolysis, real-world dataset, skin, β-Lactam antibiotics

## Abstract

**Background:**

Cutaneous adverse drug reactions (CADRs) are triggered by numerous antibiotic drugs. Most drug reactions are in the spectrum of a maculopapular eruption (MPE) with a self-limiting disease course. In contrast, severe cutaneous adverse reactions (SCARs), including Stevens–Johnson syndrome/toxic epidermal necrolysis (SJS/TEN), are rare but may follow a life-threatening course. In this study, we investigated the key drug-related triggers of CADRs in a retrospective single-center cohort and, based on these findings, evaluated the differential risk of the implicated agents using large-scale real-world data.

**Methods:**

Data from 122 patients diagnosed with CADRs, including 5 with confirmed SJS/TEN treated at a tertiary-referral hospital were retrospectively analyzed in a descriptivesingle-center cohort study. To further explore our findings, we performed a large-scale real-world cohort study using TriNetX to assess the occurrence of SJS/TEN following exposure to selected drugs. Separate analyses were conducted comparing ceftriaxone with aminopenicillins, cefazolin, cefuroxime, and allopurinol, which was included as a positive control. Odds ratios were calculated after running propensity-score-matching for demographic characteristics and SJS/TEN-risk factors.

**Results:**

In the single-center cohort, MPE was the predominant phenotype among CADRs attributed to aminopenicillins and first- and second-generation cephalosporins. In contrast, among CADRs attributed to third-generation cephalosporins, represented by ceftriaxone in this single-center cohort, SCARs were the predominant reaction type, with ceftriaxone accounting for 40% of SJS/TEN cases. These findings were underlined by the real-world cohort analysis, in which aminopenicillins and early-generation cephalosporins showed significantly lower odds of SJS/TEN than ceftriaxone (0.389 (95% CI 0.291–0.520) for aminopenicillins; 0.392 (0.290–0.529) for cefazolin; 0.300 (0.157–0.572) for cefuroxime).

**Conclusions:**

While both datasets need to be interpreted within the context of their respective limitations, our analysis, are in line with previous studies, suggesting that ceftriaxone may have an SJS/TEN risk profile that exceeds the class-wide risk observed among cephalosporins. This underscores the importance of substance-specific, rather than purely class-based approaches in both clinical research and pharmacovigilance.

## Introduction

Cutaneous adverse drug reactions (CADRs) are adverse reactions to systemic medications involving the skin, its adnexa, and mucous membranes, with clinical presentations varying widely from mild exanthematous eruptions to severe and potentially life-threatening conditions (1–3). CADRs represent a clinically important subset of type B adverse drug reactions, characterized by their dose-independent and unpredictable occurrence (1, 4). Moreover, CADRs are estimated to affect up to 10% of hospitalized patients and are often triggered by antibiotics, antiepileptics, and other drugs like pain medications such as nonsteroidal anti-inflammatory drugs (NSAIDs) (5, 6). The clinical relevance of CADRs is further underscored by an ageing population, increasing polypharmacy, and the growing complexity and intensity of pharmacotherapy in routine care, with CADRs, together with other adverse drug reactions, representing a relevant cause of hospital admissions (7–9).

The clinical course of CADRs is highly variable (1–3). Among the most common and generally mild presentations are maculopapular eruptions (MPE), which account for 30% to 95% of all CADRs (5, 10). They typically occur 4 to 14 days after the initiation of a new medication (11). In previously sensitized patients, re-exposure may lead to a more rapid recurrence, often within 1 to 4 days (11). There are also rare cases where CADRs may appear later after exposure (11). This pattern is particularly characteristic of severe cutaneous adverse reactions (SCARs), such as Stevens–Johnson syndrome/toxic epidermal necrolysis (SJS/TEN) and drug reaction with eosinophilia and systemic symptoms (DRESS), which may develop up to 4 and 8 weeks after drug exposure, respectively (11–13). In addition to SJS/TEN and DRESS, acute generalized exanthematous pustulosis (AGEP) is also classified as a SCAR, with cutaneous manifestations typically developing within 1 to 12 days after drug exposure (11, 14, 15). Although rare, SCARs comprise a clinically important subset of CADRs, representing approximately 2% to 6.7% of CADR-cases (5, 16).

While mild forms of MPE are often self-limiting, moderate cases are commonly treated with topical corticosteroids for several days. In rare cases with severe skin involvement, systemic corticosteroid therapy may be added to topical treatment (17, 18). This treatment regime is also applied to EEM-like cutaneous skin reactions, lichenoid cutaneous skin reactions, fixed drug eruption, and symmetrical drug related intertriginous and flexural exanthema (SDRIFE) (8, 19–21).

SCARs often follow a life-threatening and even fatal course, with a more complex treatment regime (1–3, 14, 15). In particular SJS/TEN and DRESS are associated with clinically relevant mortality, which is driven by the systemic involvement of internal organs (3, 22, 23). Prompt withdrawal and rapid elimination of the culprit drug plays a relevant role in the disease course and regarding SCARs (especially SJS/TEN) also survival chances (24). This underscores the importance of accurately identifying the offending substance (24).

Several high-risk substances have been identified for various types of CADRs (25, 26). In recent work, analyses of real-world data have been used to identify triggers of CADRs and to characterise their associated risk profiles (15, 27). However, available data are often restricted to broad drug classes rather than individual agents or specific subgroups of agents (27, 28). Especially in the context of antibiotic therapy, risk stratification remains a clinical concern (28). In this study we used a two-phase approach. We first conducted an exploratory analysis of a retrospective single-center cohort to identify clinically relevant triggers of CADRs, including SJS/TEN. Based on these findings, we subsequently compared the SJS/TEN risk profiles across selected β-lactams, including first to third generation cephalosporins and aminopenicillins, using real world data.

## Material and methods

In order to identify key triggers of various types of CADRs, we retrospectively analysed CADR cases treated at the inpatient dermatology department of a tertiary referral hospital (University Hospital Schleswig Holstein Campus Lübeck) from 01.03.2019 to 31.03.2021. These included MPE, AGEP, DRESS, SJS/TEN and other cutaneous skin reactions. Other cutaneous drug reactions were defined to include, EEM-like cutaneous reactions, lichenoid drug eruptions, fixed drug eruption, and baboon syndrome, also known as symmetrical drug-related intertriginous and flexural exanthema (SDRIFE). All SCAR cases were histologically confirmed by the Dermatohistopathology Unit at University Hospital Schleswig Holstein (UKSH) Campus Lübeck. Clinical classification of the SJS/TEN spectrum was based on the extent of epidermal detachment and defined as SJS with <10% detached body surface area, SJS/TEN overlap with 10%–29% detached body surface area, and TEN with ≥30% detached body surface area (29, 30). DRESS was classified using the RegiSCAR scoring system (26) and AGEP was identified according to the EuroSCAR/AGEP validation score (31). MPE and other CADR subtypes were diagnosed based on clinical presentation, disease course and additional histopathological findings in 54.9% of CADR cases.

**Figure 1:**
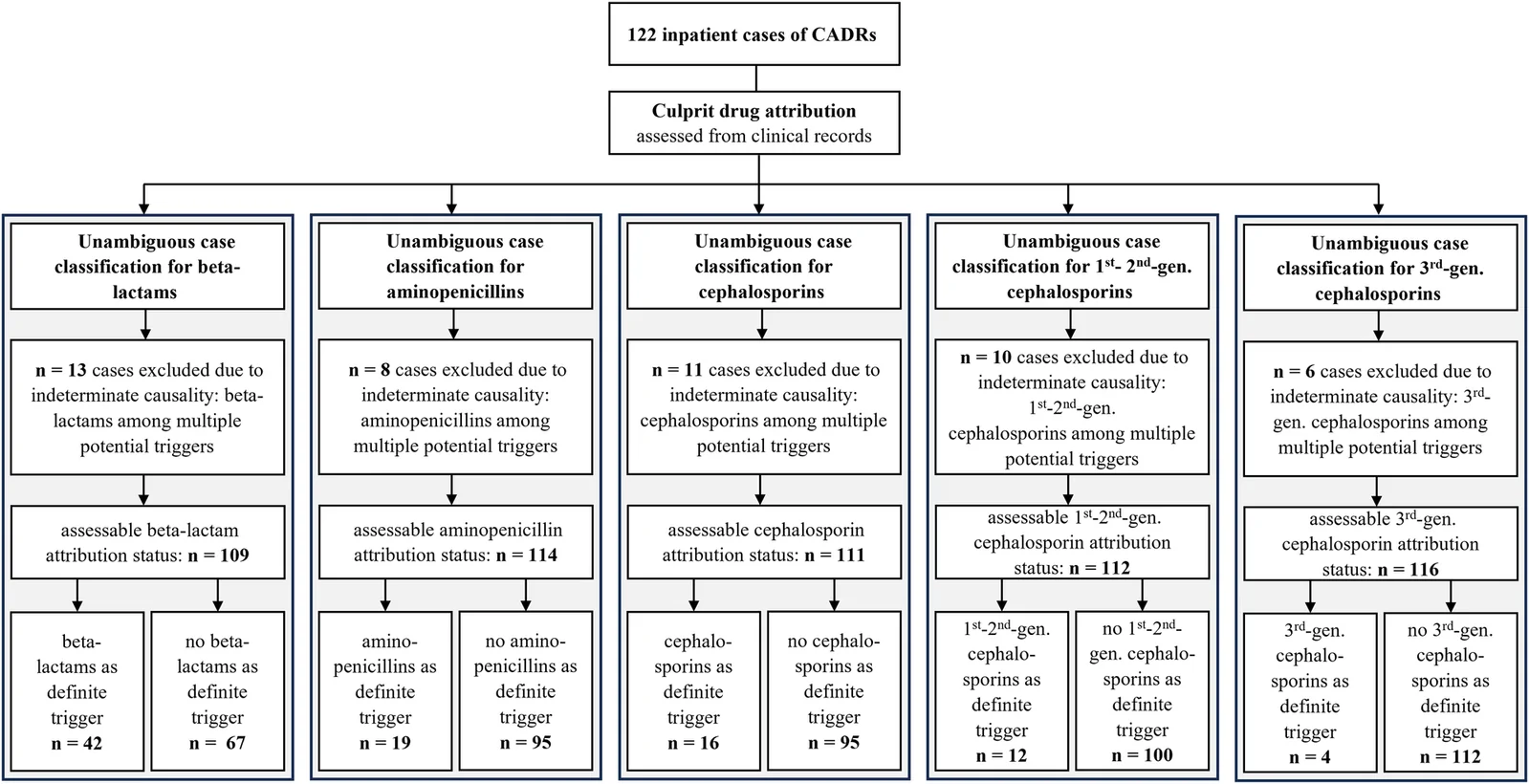
STROBE flow diagram illustrating culprit-drug attribution of beta-lactam antibiotics among CADRs in the single-center cohort. Cases with multiple potential triggers including the investigated agent were excluded for each individual agent (group) due to indeterminate causality; cases with multiple triggers not involving the investigated agent (group) were retained, resulting in varying numbers of included cases across the subsequent drug attribution assessment. CADR, cutaneous adverse drug reaction; n, number of cases; 1st-/2nd-gen., first- and second-generation; 3rd-gen., third-generation.

For this single-center CADR cohort the attributed casual triggers were evaluated descriptively. Frequencies and proportions were used to summarise identified triggers and CADR-types in the cohort, including distribution by drug class, individual agent and CADR-type. In addition, the proportion of MPE and SCARs among CADRs attributed to β-lactams, including cephalosporins and aminopenicillins, was calculated. As MPE represents the most frequent, well-characterized, and typically milder CADR phenotype, it provided a clinically meaningful descriptive comparator. This approach enabled assessment of whether selected agents showed phenotype distributions with lower proportions of MPE and higher proportions of other, potentially more severe CADR phenotypes. To maximise attribution accuracy, only cases with unambiguous causal-agent attribution were included in this agent specific analysis. Cases with multiple possible triggers involving the investigated substance were excluded. This exclusion criterion was applied when a patient had been exposed to two or more suspected drugs that were considered plausible triggers of the CADR, either because they had been newly initiated within a compatible time window before symptom onset or because the respective drug class is known to be a typical trigger of the observed CADR. In such cases, unambiguous attribution to the investigated substance was not possible, and the case was therefore excluded from the respective agent-specific summary. The selection of cases with unambiguous CADR-drug attribution is shown in the STROBE diagram (Figure 1).

**Figure 2:**
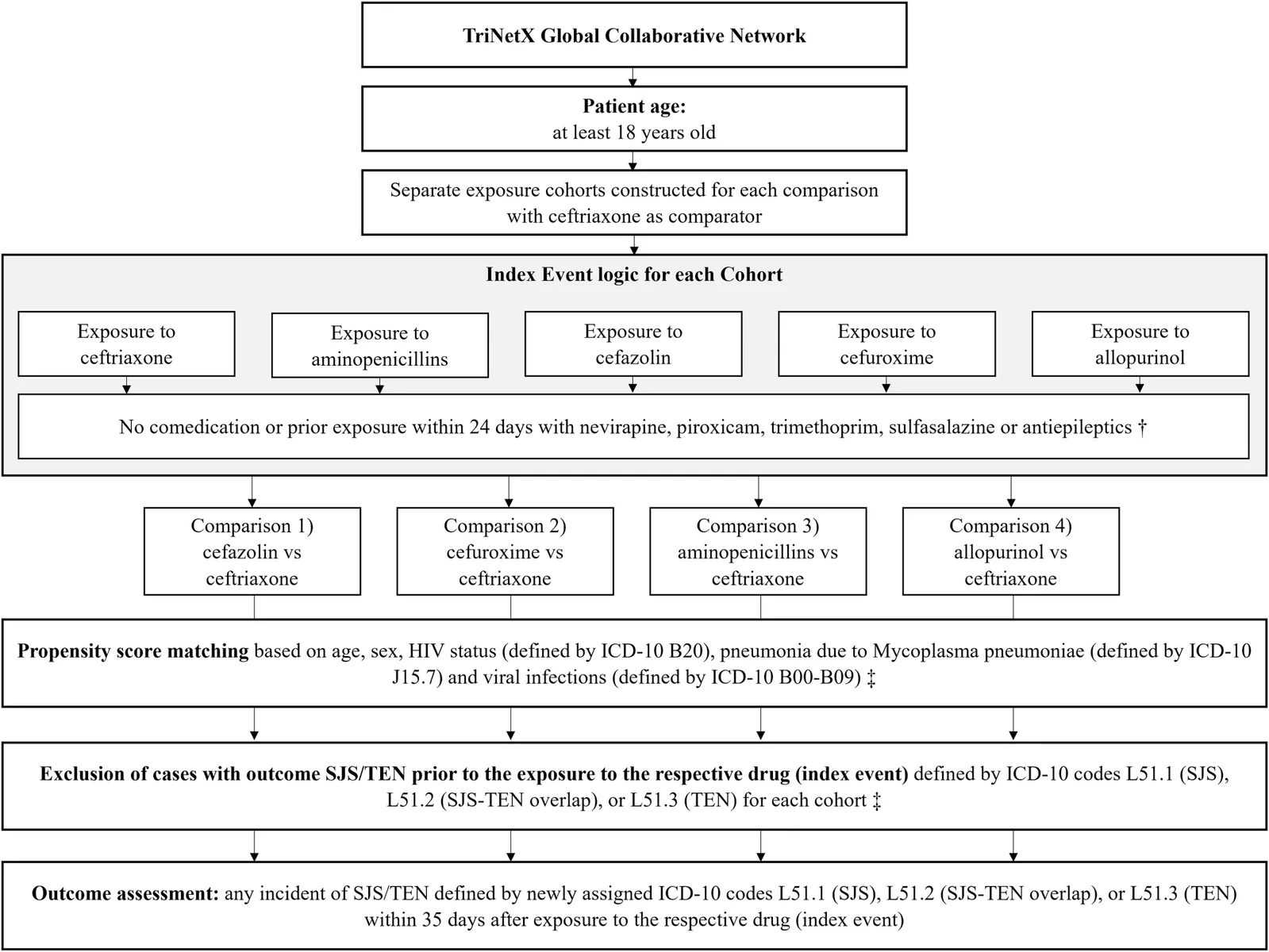
STROBE flow diagram illustrating the triNetX real-world data analysis, including query-building logic, propensity score matching, and outcome evaluation. Analyses were conducted on July 25, 2025, using the TriNetX Global Collaborative Network, incorporating data from up to 20 years prior. Excluding cases with an SJS/TEN outcome prior to the index event after running propensity score matching is inherently part of the TriNetX architecture. † Patients with exposure to nevirapine, piroxicam, trimethoprim, sulfasalazine, or antiepileptics were excluded. Substances were defined using ATC and RxNorm codes. Detailed information on the codes used for the TriNetX query can be found in Supplementary Table S5. ‡ Detailed information, including cohort sizes before and after propensity score matching and exclusion, is provided in Supplementary Tables S1–S4. ATC, Anatomical Therapeutic Chemical classification; HIV, human immunodeficiency virus; ICD-10, International Classification of Diseases, 10th Revision; SJS, Stevens-Johnson syndrome; TEN, toxic epidermal necrolysis.

To further investigate the drug-related patterns observed in our data set, particularly for selected β-lactams we conducted a large-scale real-world cohort study using TriNetX (32). We evaluated the risk of SJS/TEN following exposure to antibiotics in our single-center cohort, with a particular focus on individual cephalosporins and aminopenicillins. For each individual drug under investigation, a separate real-world cohort of patients aged at least 18 years was constructed. The STROBE diagram (Figure 2) illustrates the TriNetX query building process. We performed multiple separate analyses, comparing aminopenicillins (ampicillin/amoxicillin), a first-generation cephalosporin (cefazolin) and second-generation cephalosporin (cefuroxime) with ceftriaxone. Since ceftriaxone was the most frequently observed trigger of SJS/TEN in the single-center cohort, it was used as the reference. All analyses were performed using the TriNetX Global Collaborative Network. To support the validity of the real-world analysis and to include a positive control for the SJS/TEN outcome, we added allopurinol to the comparison, a drug with a well-established risk of inducing SJS/TEN (25). The index event for each cohort was defined as the first recorded exposure to the respective drug.

To reduce confounding by co-exposure to drugs, we ensured that patients had not been exposed to drugs with a known risk for SJS/TEN prior to the time window. These included nevirapine, piroxicam, trimethoprim, sulfasalazine, and antiepileptics (25, 27, 33). The RxNorm and ATC classification codes used to build the TriNetX queries, including those for the investigated agents, are provided in the Supplementary Table S5.

Given that SJS/TEN is associated with several risk factors, we used propensity score matching to reduce confounding when comparing cohorts exposed to the agents of interest. All cohorts were matched for baseline covariates including age, sex and potential risk factors for SJS/TEN. In this way our approach controlled for differences in the prevalence of positive HIV infection (defined by ICD-10 B20), pneumonia due to Mycoplasma pneumoniae (defined by ICD-10 J15.7) and viral infections (defined by ICD-10 B00-B09) between the compared groups (34). Baseline covariate balance before and after propensity score matching was evaluated using standardized mean differences (SMDs). Covariates with an absolute SMD <0.1 were considered adequately balanced between cohorts. The outcome analysis then assessed SJS/TEN events across the matched cohorts. SJS/TEN was required to occur within 35 days after the index event and was considered present if any of the ICD-10 codes L51.1 (SJS), L51.2 (SJS-TEN overlap), or L51.3 (TEN) was newly assigned during this period. As shown in Figure 2 patients with SJS/TEN recorded before the index event were excluded, as these events could not be attributed to exposure to the investigated substance. Also, SJS/TEN cases occurring after the predefined 35-day time window were not counted as positive outcomes. The analyses were conducted on July 25, 2025, using the TriNetX Global Collaborative Network, with data from up to 20 years prior.

ORs for SJS/TEN were derived from the observed event rates for each substance, using ceftriaxone exposure as the reference, and were presented in a forest plot.

## Results

As shown in Table 1 the 122 CADR cases treated at UKSH Campus Lübeck included among 81 patients diagnosed with MPE, 13 patients with AGEP, 2 patients with DRESS and 5 patients with SJS/TEN. Furthermore 21 patients were grouped into other cutaneous skin reactions. The major drug class causing CADRs was antibiotics (*n* = 63), followed by analgesics/NSAIDs (*n* = 8), antihypertensives/diuretics (*n* = 5), and antiepileptics (*n* = 3) (Table 2). Further analyses of causative drugs by CADR type showed that MPE was most commonly associated with antibiotics (*n* = 52), particularly aminopenicillins (*n* = 17) and cephalosporins (*n* = 13) (Table 3). In AGEP, the spectrum of causative agents was more heterogeneous, including both antibiotics (*n* = 3) and various non-antibiotic drugs (*n* = 6) (Table 4). In contrast, in SJS/TEN and DRESS, antibiotics were also observed to be the predominant causative agents (Table 4).

**Table 1:** Overview of CADR entities in local retrospective cohort.

Type of CADR	Frequency (n)	Percent (%)
MPE	81	66.4
AGEP	13	10.7
SJS/TEN	5	4.1
DRESS syndrome	2	1.6
Other CADRs^a^	21	17.2
Total	122	100.0

a Other CADRs comprised the following entities: EEM-like (*n* = 6), lichenoid drug reaction (*n* = 5), fixed drug eruption (*n* = 5), Symmetrical drug-related intertriginous and flexural exanthema (*n* = 5).

CADR, cutaneous adverse drug reaction; MPE, maculopapular exanthema; AGEP, acute generalized exanthematous pustulosis; SJS, Stevens–Johnson syndrome; TEN, toxic epidermal necrolysis; DRESS, drug reaction with eosinophilia and systemic symptoms; EEM, erythema exsudativum multiforme.

**Table 2:** Summary of main causative agents for all CADRs in local retrospective cohort.

Category	Frequency (n)	Percent (%)
Antibiotics^a^	63	51.6
Analgesics/NSAIDs^b^	8	6.6
Antihypertensives/Diuretics	5	4.1
Antiepileptics	3	2.5
All other agents	43	35.2
Total	122	100

Multi-agent cases were assigned to a category only if all candidate agents belonged to that category; otherwise, they were counted under “All other agents”.

a Includes antibiotic combinations that contain a β-lactamase inhibitor (e.g., amoxicillin/clavulanic acid, ampicillin/sulbactam, piperacillin/tazobactam).

b Defined as acetylsalicylic acid, ibuprofen, diclofenac, celecoxib, metamizole.

CADR, cutaneous adverse drug reaction; NSAID, non-steroidal anti-inflammatory drug.

**Table 3:** Causative agents of MPE in local retrospective cohort.

Agent class				*n*	% of MPE
Antibiotics^a^				52	64.2
	β-lactams^a^			36	44.4
		Penicillins^a^		22	27.2
			Aminopenicillins^a^	17	21.0
			Penicillin V	1	1.2
			Piperacillin^a^	4	4.9
		Cephalosporins^b^		13	16.0
			1st and 2nd gen. cephalosporins	12	14.8
			3rd gen. cephalosporins	1	1.2
		Multiple β-lactams as possible trigger		1	1.2
	Non-β-lactam antibiotics			15	18.5
		Clindamycin		10	12.3
		Other non-β-lactam antibiotics		5	6.2
	Multiple antibiotics as possible trigger			1	1.2
Non-antibiotic agents				23	28.4
	Pain killers/analgesics^c^			3	3.7
	Antihypertensives/diuretics^c^			3	3.7
	Antiepileptics			2	2.5
	Other non-antibiotic agents			15	18.5
Multiple possible antibiotic/non-antibiotic triggers				6	7.4
Total MPE				81	100.0

a Includes antibiotic combinations that contain a β-lactamase inhibitor (e.g., amoxicillin/clavulanic acid, ampicillin/sulbactam, piperacillin/tazobactam).

b First- and second-generation cephalosporins included cefazolin and cefuroxime; third-generation cephalosporins included ceftriaxone.

c Defined as acetylsalicylic acid, ibuprofen, diclofenac, celecoxib or metamizole.

MPE, maculopapular exanthema.

**Table 4:** Causative agents of SCARs (AGEP, SJS/TEN, DRESS) in local retrospective cohort.

SCAR type			*n*	% of CADRs	% within SCAR-type
AGEP	Total		13	9.6	100
	Antibiotics		3	2.2	23.1
		Ceftriaxone	1	0.7	7.7
		Ciprofloxacin	1	0.7	7.7
		Linezolid	1	0.7	7.7
	Non-antibiotic agents		6	4.4	46.2
		Celecoxib	1	0.7	7.7
		Everolimus	1	0.7	7.7
		Itraconazole	1	0.7	7.7
		Metamizole	3	2.2	23.1
	Multiple possible triggers^a^		4	3	30.8
SJS/TEN	Total		5	3.7	100
		Ceftriaxone	2	1.5	40
		Lamotrigine	1	0.7	20
		Multiple possible triggers^a^	2	1.5	40
DRESS	Total		2	1.5	100
		Amoxicillin	1	0.7	50
		Multiple possible triggers^a^	1	0.7	50

a multiple possible triggers: more than one potential causative agent was considered causal.

SCAR, severe cutaneous adverse reaction; AGEP, acute generalized exanthematous pustulosis; SJS, Stevens–Johnson syndrome; TEN, toxic epidermal necrolysis; DRESS, drug reaction with eosinophilia and systemic symptoms; CADR, cutaneous adverse drug reaction.

To further characterize the CADR spectrum attributed to β-lactams, particularly cephalosporins and aminopenicillins, an agent-specific summary is presented in Table 5. This summary included only cases in which it could be unambiguously determined whether the CADR was attributed to one of the listed agents or not. Cases with multiple potential triggers involving the respective drug or drug group under investigation were excluded from the corresponding agent-specific summary, as shown in Figure 1. Accordingly, 13 CADR cases were excluded from the beta-lactam assessment, 8 from the aminopenicillin assessment, 11 from the cephalosporin assessment, 10 from the first- and second-generation cephalosporin assessment, and 6 from the third-generation cephalosporin assessment. Across all CADRs attributed to β-lactams, MPE accounted for 85.7% of cases, whereas SCARs accounted for 9.5%. CADRs attributed to aminopenicillins showed a phenotype distribution, with most cases presenting as MPE (89.5%) and only a small proportion classified as SCARs (5.3%). In contrast, CADRs attributed to cephalosporins showed a higher proportion of SCARs (18.8%). However, within the subgroup of first- and second-generation cephalosporins, MPE was observed in all CADRs attributed to cefazolin and cefuroxime, whereas among CADRs attributed to third-generation cephalosporins, represented by ceftriaxone in this cohort, SCARs were the predominant reaction type, with MPE accounting for only one of four CADRs. As presented in Table 4, ceftriaxone was the most frequent trigger of SJS/TEN.

**Table 5:** Number of CADRs attributed to each agent in the single-center retrospective cohort, including the proportion of MPE and SCARs.

Investigated agent^a^	CADRs due to agent, n	Proportion of MPE in CADRs due to agent (%)	Proportion of SCARs in CADRs due to agent (%)
all Beta-lactams	42	85.7	9.5
Aminopenicillins	19	89.5	5.3
all Cephalosporins	16	81.3	18.8
1st- and 2nd-Generation Cephalosporins	12	100.0	0.0
3rd-Generation Cephalosporins	4	25.0	75.0

Only cases with unambiguous causal-agent attribution were included in the analysis; cases with multiple possible triggers involving the investigated substance were excluded.

a Beta-lactams included penicillin V, aminopenicillins, and piperacillin; aminopenicillins included amoxicillin and ampicillin; first- and second-generation cephalosporins included cefazolin and cefuroxime; third-generation cephalosporins included ceftriaxone.

CADR, cutaneous adverse drug reaction; MPE, maculopapular exanthema; SCAR, severe cutaneous adverse reaction.

**Figure 3:**
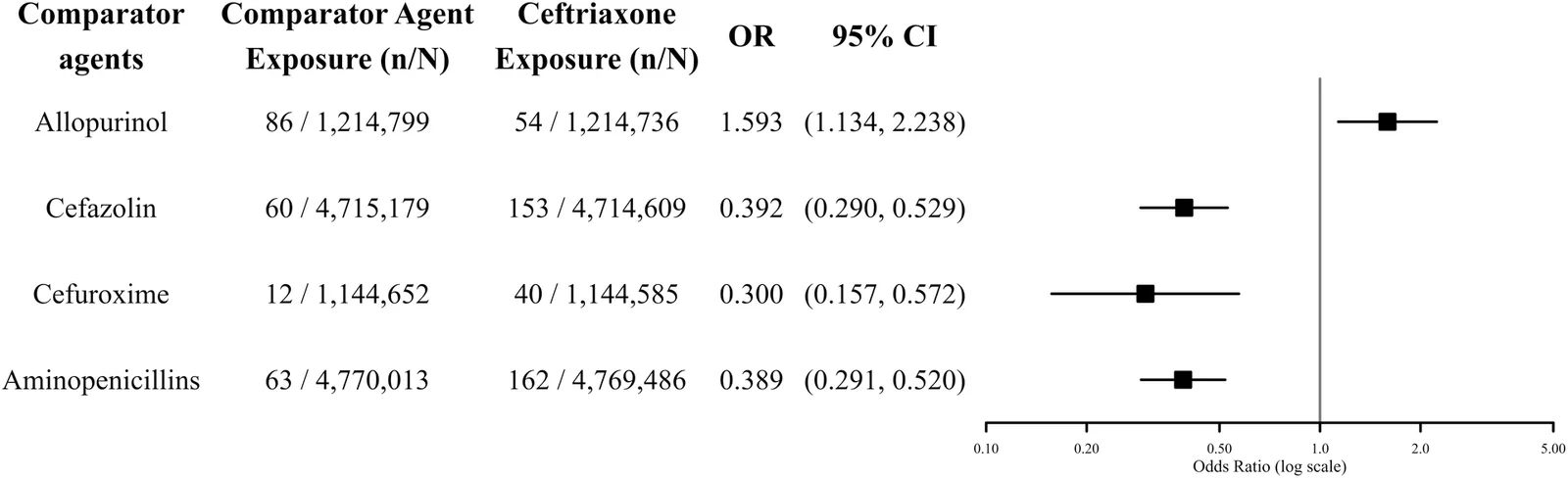
Real-world data analysis. Forest plot showing odds ratios and 95% confidence intervals for reactions of the Stevens-Johnson syndrome/toxic epidermal necrolysis spectrum (SJS/TEN) occurring within 35 days of exposure to each tested drug compared with ceftriaxone as reference drug. SJS/TEN cases were defined by a newly assigned ICD- code (L51.1, L51.2, or L51.3). Patients with exposure to nevirapine, piroxicam, trimethoprim, sulfasalazine, or antiepileptics (defined by ATC code N03) were excluded. Cohorts were matched using propensity-score matching for age, sex, HIV status (defined by ICD-10 B20), pneumonia due to Mycoplasma pneumoniae (defined by ICD-10 J15.7) and viral infections (defined by ICD-10 B00-B09). Analyses were conducted on July 25, 2025, using the TriNetX Global Collaborative Network, with data from up to 20 years prior. CI, confidence interval; n/N, case counts among exposed; OR, odds ratio; ATC, Anatomical Therapeutic Chemical classification.

Based on the findings of this single-center study, we further assessed the risk of SJS/TEN after antibiotic exposure using TriNetX. The baseline characteristics of the TriNetX cohorts before and after propensity score matching are presented in Supplementary Tables S1–S4, demonstrating adequate post-matching balance across all assessed covariates, with absolute standardized mean differences below 0.1. The results of the large-scale real-world analysis are presented in the forest plot (Figure 3). The third-generation cephalosporin ceftriaxone was used as the comparator in the analyses and is represented by the vertical line in Figure 3. Across the four analyses, 1,214,799 allopurinol-exposed, 4,715,179 cefazolin-exposed, 1,144,652 cefuroxime-exposed, and 4,770,013 aminopenicillin-exposed patients were identified and matched to corresponding ceftriaxone-exposed cohorts of similar size, as shown in Figure 3.

Compared with ceftriaxone, aminopenicillins, cefazolin, and cefuroxime were all associated with significantly lower odds of SJS/TEN. The corresponding odds ratios were 0.389 (95% CI 0.291–0.520) for aminopenicillins, 0.392 (95% CI 0.290–0.529) for cefazolin, and 0.300 (95% CI 0.157–0.572) for cefuroxime. The positive control cohort, which was exposed to allopurinol, showed the highest observed odds of SJS/TEN compared with ceftriaxone.

## Discussion

While other studies show a significant association between cephalosporins and SJS/TEN, they do not distinguish individual agents (27, 28, 35). However, a retrospective study provided an agent-level breakdown of antibiotic-associated SJS/TEN cases (*n* = 75) and showed that 92.3% of cephalosporin-triggered SJS/TEN (*n* = 26) involved third-generation agents (*n* = 24), the majority of which were related to ceftriaxone (36). Accordingly, a large pharmacovigilance study using the FDA adverse event reporting system evaluated SCAR signals across multiple culprit drugs, including multiple β-lactam antibiotics (15). In that analysis, ceftriaxone showed higher reporting rates and stronger disproportionality signals for TEN and SJS/TEN overlap than aminopenicillins, cefazolin and cefuroxime, indicating a comparatively stronger association with these SCAR phenotypes (15). Although spontaneous reporting data cannot provide incidence estimates and are subject to reporting bias, the pharmacovigilance findings were consistent with our observations across both datasets.

In our single-center cohort, ceftriaxone accounted for 40% of SJS/TEN cases. In contrast, MPE, representing the milder end of the CADR spectrum due to its higher frequency and generally benign clinical course, was predominantly observed among CADRs attributed to aminopenicillins and early-generation cephalosporins. In the real-world analysis, the effect estimates for cefazolin, cefuroxime, and aminopenicillins were within a comparable range and consistently indicated lower odds of SJS/TEN compared with ceftriaxone, thereby supporting the pattern of the descriptive single-center observations. Although ceftriaxone showed higher odds than the antibiotic comparators, they remained below those of allopurinol, confirming the known high SJS/TEN risk of the latter. Taken together, these findings may point toward differences in the CADR spectrum and SJS/TEN risk profiles among individual β-lactams, particularly within the cephalosporin class.

Nevertheless, both our analysis settings (real-world study, single-center study) have limitations. Because our single-center study had a retrospective design, the frequency with which individual drugs are prescribed in clinical practice may have influenced the number of reactions observed for each drug. Due to the rarity of SCARs, the number of observed severe reactions in the single-center cohort was limited, reducing the robustness of conclusions based on this cohort alone. However, interpreted in the context of the literature described above, these findings provided a rationale for the subsequent real-world data analysis. Furthermore, the lack of denominator data in the local cohort and the potential for selection bias due to the exclusion of cases with multiple possible triggers represent additional limitations to consider when interpreting the descriptive findings.

Our real-world analysis contributes a more controlled approach by accounting for demographic factors and established risk factors for SJS/TEN through propensity score matching. However, as it is based on routinely collected electronic health record data, it may be subject to coding inaccuracies, incomplete documentation, and residual confounding. Moreover, confounding by indication must be considered, since ceftriaxone is generally given to sicker individuals, meaning the results could potentially reflect illness severity, inpatient status, infection type, sepsis, intensive care unit care, polypharmacy, renal disease, malignancy, or concomitant drugs rather than ceftriaxone itself. Lastly, potential misclassification of SJS/TEN by ICD codes constitute limitations to consider.

Overall, our large-scale real-world analysis showed that ceftriaxone occupied an intermediate risk position, lying between allopurinol and the other β-lactam antibiotics included in the comparison (Figure 3). Consistent with this, other pharmacovigilance studies have shown that ceftriaxone is more frequently reported in association with SJS/TEN compared to other antibiotic agents (15, 36). Although disease severity could not be fully captured in our data, our risk factor–adjusted approach accounted for several relevant patient-level covariates. This, together with the observed signal, may suggest that ceftriaxone, as a third-generation cephalosporin, carries an SJS/TEN risk profile that differs from, and may exceed, the underlying class-wide risk among cephalosporins. These findings highlight the need for agent-level rather than class-level pharmacovigilance, as the risk of SJS/TEN may not be uniform across individual agents within the cephalosporin class and may more broadly differ across β-lactams. Further multicenter studies controlling for disease severity, indication, and comorbidity burden are needed to clarify the clinical implications of these findings

## Data Availability

The original contributions presented in the study are included in the article/Supplementary Material, further inquiries can be directed to the corresponding author/s.
