# Supplementary material for: Differential risk signals of cutaneous adverse drug reactions among β-lactam antibiotics: ceftriaxone shows increased signals for SJS/TEN in clinical and real-world cohorts
Source: Front Med (Lausanne). 2026 Sep 3;13:1875934. doi: 10.3389/fmed.2026.1875934 (PMC13581707; doi:10.3389/fmed.2026.1875934)
Supplement: Supplementary file 1 [file Table1.docx]

**Supplementary Tables:**

Supplementary Table 1: **Baseline characteristics of cefazolin and ceftriaxone cohorts before and after propensity score matching**

Supplementary Table 2: **Baseline characteristics of cefuroxime and ceftriaxone cohorts before and after propensity score matching**

Supplementary Table 3: **Baseline characteristics of aminopenicillins and ceftriaxone cohorts before and after propensity score matching**

Supplementary Table 4: **Baseline characteristics of allopurinol and ceftriaxone cohorts before and after propensity score matching**

Supplementary Table 5: **Definition of drug exposure concepts used for TriNetX query building**

**Supplementary Table 1 Baseline characteristics of cefazolin and ceftriaxone cohorts before and after propensity score matching**

| **Characteristic** | **Cefazolin before PSM** | **Ceftriaxone before PSM** | **p-value before PSM** | **SMD before PSM** | **Cefazolin after PSM** | **Ceftriaxone after PSM** | **p-value after PSM** | **SMD after PSM** | **Cefazolin cases excluded (SJS/TEN prior to index exposure)** | **Ceftriaxone cases excluded (SJS/TEN prior to index exposure)** | **Cefazolin final cohort** | | **Ceftriaxone final  cohort** |
| --- | --- | --- | --- | --- | --- | --- | --- | --- | --- | --- | --- | --- | --- |
| **Evaluable patients, N** | 9,652,884 | 4,846,326 |  |  | 4,715,743 | 4,715,743 |  |  | 564 | 1,134 | 4,715,179 | | 4,714,609 |
| Age at index, mean ± SD | 50.9 ± 19.8 | 53.1 ± 22.8 | <0.001 | 0.105 | 52.7 ± 22.5 | 52.7 ± 22.6 | 0.001 | 0.002 |  |  |  |  | |
| Female, % | 53.638 | 52.735 | <0.001 | 0.018 | 51.958 | 52.909 | <0.001 | 0.019 |  |  |  |  | |
| Male, % | 43.052 | 44.445 | <0.001 | 0.028 | 44.541 | 44.321 | <0.001 | 0.004 |  |  |  |  | |
| Human immunodeficiency virus [HIV] disease, B20, % | 0.142 | 0.439 | <0.001 | 0.055 | 0.290 | 0.290 | 1.000 | <0.001 |  |  |  |  | |
| Other viral diseases, B25–B34, % | 0.142 | 1.449 | <0.001 | 0.147 | 0.291 | 0.288 | 0.380 | 0.001 |  |  |  |  | |
| Pneumonia due to *Mycoplasma pneumoniae*, J15.7, % | 0.002 | 0.046 | <0.001 | 0.028 | 0.004 | 0.007 | <0.001 | 0.004 |  |  |  |  | |

Values are presented as mean ± standard deviation for continuous variables and as percentages for categorical variables. Percentages were calculated using the number of evaluable patients in each cohort. For sex, percentages may not sum to 100% because patients without an assigned sex label in TriNetX were not reported as a separate category. ICD-10 codes listed for diagnosis characteristics indicate the diagnostic codes used to define the respective characteristics. Covariate balance before and after propensity score matching was assessed using standardized mean differences, with absolute values <0.1 indicating adequate balance. P-values are provided descriptively. ICD-10, International Classification of Diseases, 10th Revision; PSM, propensity score matching; SMD, standardized mean difference.

**Supplementary Table 2 Baseline characteristics of cefuroxime and ceftriaxone cohorts before and after propensity score matching**

| **Characteristic** | **Cefuroxime before PSM** | **Ceftriaxone before PSM** | **p-value before PSM** | **SMD before PSM** | **Cefuroxime after PSM** | **Ceftriaxone after PSM** | **p-value after PSM** | **SMD after PSM** | **Cefuroxime cases excluded (SJS/TEN prior to index exposure)** | **Ceftriaxone cases excluded (SJS/TEN prior to index exposure)** | **Cefuroxime final cohort** | **Ceftriaxone final cohort** |
| --- | --- | --- | --- | --- | --- | --- | --- | --- | --- | --- | --- | --- |
| **Evaluable patients, N** | 1,144,887 | 4,846,326 |  |  | 1,144,887 | 1,144,887 |  |  | 235 | 302 | 1,144,652 | 1,144,585 |
| Age at index, mean ± SD | 56.7 ± 20.5 | 53.1 ± 22.8 | <0.001 | 0.166 | 56.7 ± 20.5 | 56.7 ± 20.5 | 1.000 | <0.001 |  |  |  |  |
| Female, % | 56.696 | 52.735 | <0.001 | 0.080 | 56.696 | 56.696 | 1.000 | <0.001 |  |  |  |  |
| Male, % | 40.288 | 44.445 | <0.001 | 0.084 | 40.288 | 40.741 | <0.001 | 0.009 |  |  |  |  |
| Human immunodeficiency virus [HIV] disease, B20, % | 0.119 | 0.439 | <0.001 | 0.061 | 0.119 | 0.119 | 0.985 | <0.001 |  |  |  |  |
| Other viral diseases, B25–B34, % | 0.695 | 1.449 | <0.001 | 0.073 | 0.695 | 0.695 | 0.994 | <0.001 |  |  |  |  |
| Pneumonia due to *Mycoplasma pneumoniae*, J15.7, % | 0.013 | 0.046 | <0.001 | 0.019 | 0.013 | 0.013 | 0.954 | <0.001 |  |  |  |  |

Values are presented as mean ± standard deviation for continuous variables and as percentages for categorical variables. Percentages were calculated using the number of evaluable patients in each cohort. For sex, percentages may not sum to 100% because patients without an assigned sex label in TriNetX were not reported as a separate category. ICD-10 codes listed for diagnosis characteristics indicate the diagnostic codes used to define the respective characteristics. Covariate balance before and after propensity score matching was assessed using standardized mean differences, with absolute values <0.1 indicating adequate balance. P-values are provided descriptively. ICD-10, International Classification of Diseases, 10th Revision; PSM, propensity score matching; SMD, standardized mean difference.

**Supplementary Table 3 Baseline characteristics of aminopenicillins and ceftriaxone cohorts before and after propensity score matching**

| **Characteristic** | **Amino-penicillins before PSM** | **Ceftriaxone before PSM** | **p-value before PSM** | **SMD before PSM** | **Amino-penicillins after PSM** | **Ceftriaxone after PSM** | **p-value after PSM** | **SMD after PSM** | **Aminopenicillins cases excluded (SJS/TEN prior to index exposure)** | **Ceftriaxone cases excluded (SJS/TEN prior to index exposure)** | **Amino-penicillins final cohort** | **Ceftriaxone final cohort** |
| --- | --- | --- | --- | --- | --- | --- | --- | --- | --- | --- | --- | --- |
| **Evaluable patients, N** | 12,100,912 | 4,811,027 |  |  | 4,770,652 | 4,770,652 |  |  | 639 | 1,166 | 4,770,013 | 4,769,486 |
| Age at index, mean ± SD | 45.0 ± 21.7 | 53.1 ± 22.8 | <0.001 | 0.363 | 52.9 ± 22.7 | 52.9 ± 22.8 | <0.001 | 0.002 |  |  |  |  |
| Female, % | 55.284 | 52.730 | <0.001 | 0.051 | 52.346 | 52.820 | <0.001 | 0.009 |  |  |  |  |
| Male, % | 42.308 | 44.429 | <0.001 | 0.043 | 44.835 | 44.356 | <0.001 | 0.010 |  |  |  |  |
| Human immunodeficiency virus [HIV] disease, B20, % | 0.145 | 0.435 | <0.001 | 0.054 | 0.368 | 0.334 | <0.001 | 0.006 |  |  |  |  |
| Other viral diseases, B25–B34, % | 0.689 | 1.447 | <0.001 | 0.074 | 1.275 | 1.244 | <0.001 | 0.003 |  |  |  |  |
| Pneumonia due to *Mycoplasma pneumoniae*, J15.7, % | 0.013 | 0.046 | <0.001 | 0.020 | 0.029 | 0.043 | <0.001 | 0.007 |  |  |  |  |

Values are presented as mean ± standard deviation for continuous variables and as percentages for categorical variables. Percentages were calculated using the number of evaluable patients in each cohort. For sex, percentages may not sum to 100% because patients without an assigned sex label in TriNetX were not reported as a separate category. ICD-10 codes listed for diagnosis characteristics indicate the diagnostic codes used to define the respective characteristics. Covariate balance before and after propensity score matching was assessed using standardized mean differences, with absolute values <0.1 indicating adequate balance. P-values are provided descriptively. ICD-10, International Classification of Diseases, 10th Revision; PSM, propensity score matching; SMD, standardized mean difference.

**Supplementary Table 4 Baseline characteristics of allopurinol and ceftriaxone cohorts before and after propensity score matching**

| **Characteristic** | **Allopurinol before PSM** | **Ceftriaxone before PSM** | **p-value before PSM** | **SMD before PSM** | **Allopurinol after PSM** | **Ceftriaxone after PSM** | **p-value after PSM** | **SMD after PSM** | **Allopurinol cases excluded (SJS/TEN prior to index exposure)** | **Ceftriaxone cases excluded (SJS/TEN prior to index exposure)** | **Allopurinol final cohort** | **Ceftriaxone final cohort** |
| --- | --- | --- | --- | --- | --- | --- | --- | --- | --- | --- | --- | --- |
| **Evaluable patients, N** | 1,215,047 | 4,846,326 |  |  | 1,215,047 | 1,215,047 |  |  | 248 | 311 | 1,214,799 | 1,214,736 |
| Age at index, mean ± SD | 64.8 ± 14.8 | 53.1 ± 22.8 | <0.001 | 0.607 | 64.8 ± 14.8 | 64.8 ± 14.8 | 0.996 | <0.001 |  |  |  |  |
| Female, % | 27.347 | 52.735 | <0.001 | 0.536 | 27.347 | 27.347 | 0.999 | <0.001 |  |  |  |  |
| Male, % | 69.999 | 44.445 | <0.001 | 0.535 | 69.999 | 68.318 | <0.001 | 0.036 |  |  |  |  |
| Human immunodeficiency virus [HIV] disease, B20, % | 0.181 | 0.439 | <0.001 | 0.046 | 0.181 | 0.181 | 0.988 | <0.001 |  |  |  |  |
| Pneumonia due to *Mycoplasma pneumoniae*, J15.7, % | 0.065 | 0.061 | 0.086 | 0.002 | 0.065 | 0.020 | <0.001 | 0.022 |  |  |  |  |
| Other viral diseases, B25–B34, % | 0.031 | 0.018 | <0.001 | 0.008 | 0.031 | 0.004 | <0.001 | 0.020 |  |  |  |  |

Values are presented as mean ± standard deviation for continuous variables and as percentages for categorical variables. Percentages were calculated using the number of evaluable patients in each cohort. For sex, percentages may not sum to 100% because patients without an assigned sex label in TriNetX were not reported as a separate category. ICD-10 codes listed for diagnosis characteristics indicate the diagnostic codes used to define the respective characteristics. Covariate balance before and after propensity score matching was assessed using standardized mean differences, with absolute values <0.1 indicating adequate balance. P-values are provided descriptively. ICD-10, International Classification of Diseases, 10th Revision; PSM, propensity score matching; SMD, standardized mean difference.

**Supplementary Table 5**

**Definition of drug exposure concepts used for TriNetX query building**

| **Substance / drug class** | **TriNetX concept code** |
| --- | --- |
| Allopurinol | NLM:RXNORM:519 |
| Amoxicillin | NLM:RXNORM:723 |
| Ampicillin | NLM:RXNORM:733 |
| Antiepileptics | NLM:ATC:N03 |
| Cefazolin | NLM:RXNORM:2180 |
| Ceftriaxone | NLM:RXNORM:2193 |
| Cefuroxime | NLM:RXNORM:2194 |
| Nevirapine | NLM:RXNORM:53654 |
| Piroxicam | NLM:RXNORM:8356 |
| Sulfasalazine | NLM:RXNORM:9524 |
| Trimethoprim and derivatives | NLM:ATC:J01EA |

RxNorm and ATC classification codes were used to
define drug exposure concepts for TriNetX query building.
